# Investigating Surface and Near-Surface Bushfire Fuel Attributes: A Comparison between Visual Assessments and Image-Based Point Clouds

**DOI:** 10.3390/s17040910

**Published:** 2017-04-20

**Authors:** Christine Spits, Luke Wallace, Karin Reinke

**Affiliations:** 1 School of Science, RMIT University, Melbourne 3001, Australia; luke.wallace2@rmit.edu.au (L.W.); karin.reinke@rmit.edu.au (K.R.); 2 Bushfire and Natural Hazards Cooperative Research Centre, East Melbourne 3002, Australia

**Keywords:** image-based point clouds, fuel structure, visual assessment

## Abstract

Visual assessment, following guides such as the Overall Fuel Hazard Assessment Guide (OFHAG), is a common approach for assessing the structure and hazard of varying bushfire fuel layers. Visual assessments can be vulnerable to imprecision due to subjectivity between assessors, while emerging techniques such as image-based point clouds can offer land managers potentially more repeatable descriptions of fuel structure. This study compared the variability of estimates of surface and near-surface fuel attributes generated by eight assessment teams using the OFHAG and Fuels3D, a smartphone method utilising image-based point clouds, within three assessment plots in an Australian lowland forest. Surface fuel hazard scores derived from underpinning attributes were also assessed. Overall, this study found considerable variability between teams on most visually assessed variables, resulting in inconsistent hazard scores. Variability was observed within point cloud estimates but was, however, on average two to eight times less than that seen in visual estimates, indicating greater consistency and repeatability of this method. It is proposed that while variability within the Fuels3D method may be overcome through improved methods and equipment, inconsistencies in the OFHAG are likely due to the inherent subjectivity between assessors, which may be more difficult to overcome. This study demonstrates the capability of the Fuels3D method to efficiently and consistently collect data on fuel hazard and structure, and, as such, this method shows potential for use in fire management practices where accurate and reliable data is essential.

## 1. Introduction

Fire is a fundamental process for maintaining diversity and health in Australian ecosystems [1,2]. However, uncontrolled bushfires can pose a significant risk to human life and property [3,4], and result in substantial economic losses and environmental impacts [5]. Fire risk is strongly related to properties of vegetation, such as the quantity, structure and orientation, the portion of live and dead biomass, and moisture content [6]. Quantifying and characterising vegetation as fuel is therefore essential to understanding fire behaviour [7], particularly as fuel is the only factor influencing fire behaviour that can be manipulated by land managers [8]. Understanding fuel also helps to inform a wide range of fire management activities such as assessing bushfire risk, planning fuel treatments, and managing smoke emissions [8,9,10,11,12].

Classifying and quantifying fuel is complex and includes assessing a range of characteristics, such as fine fuel load, live-to-dead ratio, height, bulk density, vertical and horizontal connectivity, and the quantity of individual fuel layers [11,13]. Of these, fine fuel load (<6 mm diameter) has been considered one of the most significant fuel variables affecting the behaviour of fire [14,15] and has been used to predict the rate of fire spread [7,16]. However, more recent models by Cheney et al. [17] and other studies such Kreye et al. [18] indicate that fuel hazard and other attributes may have stronger associations with rate of spread than fuel alone. Furthermore, the structure and arrangement of fuel types within the fuel profile is also a key factor determining fire behaviour [16,19,20].

Assessment of fuels and their respective “hazard” rating is commonly based on visual field assessments of the different fuel layers and associated descriptive variables. Methods for fuel assessment used in Australia include the Overall Fuel Hazard Assessment Guide (OFHAG, [21]), which is utilised by most of the eastern states and forms the basis of similar guides in other states (see [22,23]). These assessment methods aim to attribute hazard ratings to vegetation based on the structure and continuity of fuels, the live-to-dead ratio, height and size for each of the four fuel layers i.e., surface litter, near-surface fuel, elevated (shrub) fuels and bark [9,22].

Visual estimation of fuel attributes provides a low-cost and efficient method of rapidly characterising fuels and individual fuel layers compared to direct measurement techniques (e.g., destructive sampling), which can be laborious and time-consuming [24]. However, visual assessments are subjective and can be vulnerable to inconsistency due to variability between assessors [24,25,26]. For example, observer estimates of vegetation condition parameters such as cover and height have been found to vary considerably between observers using a range of visual assessment methods, including BioCondition [27] and Habitat Hectares [28]. With respect to fuels, Sikkink and Keane [25] found that visual fuel load estimates varied when comparing several local assessment methods in the United States. Within Australia, a study looking at the third edition of the OFHAG [29] and Project Vesta [16,22] found significant differences between teams on most variables assessed, including final fuel hazard ratings [24]. In particular, the study undertaken by Watson et al. [24] found high variability in the response from assessors on individual parameters (e.g., percent cover, height) for surface, near-surface and elevated fuels, and individual component fuel hazard ratings. Furthermore, a recent study looking at forest fuel loads using the OFHAG found little correlation between visually assessed fuel hazard scores and destructively sampled biomass, with assessors tending to overestimate fuel hazard at low fuel loads and underestimate hazard at high fuel loads, regardless of fuel type [30].

Other methods of indirectly assessing fuel hazard have emerged in recent years, such as remote sensing and photogrammetry. Recent studies have demonstrated the potential of satellite and airborne remote sensing for assessing and monitoring vegetation (fuel) structure [31,32,33,34], while Gupta et al. [35] have demonstrated that Terrestrial Laser Scanning (TLS) is an effective tool in assessing temporal changes in surface and near-surface fuels. Studies utilising Light Detection and Ranging (LiDAR) systems have also demonstrated that this technology is effective at measuring canopy and surface fuel parameters [36,37]. However, technologies such as LiDAR and TLS can be cost prohibitive [38], and are therefore limited in their application. Recent studies have demonstrated the value of more cost-effective methods of capturing information describing vegetation (fuel) structure and health using image-based point clouds [39,40,41,42,43,44,45]. Image based point clouds are constructed using photogrammetric principles from highly overlapping photography, and the reader is directed to Westoby et al. [46] and Snavely et al. [47] for further details on the methods. Liang et al. [42], for example, demonstrated that terrestrial point clouds from a handheld camera were capable of measuring tree attributes with low cost, high efficiency and acceptable accuracy, while a case study undertaken by Wallace et al. [48] demonstrates the potential for imagery captured on smartphones to adequately reconstruct the 3D structure of grassland environments. Furthermore, a recent study looking at the utility of imagery captured on a handheld camera to accurately estimate vegetation structure found a high correlation between destructively sampled biomass and vegetation volume derived from image-based point clouds in pasture (*r*^2^ = 0.78), dry grassy forest (*r*^2^ = 0.87) and lowland forest (*r*^2^ = 0.63) [40]. Studies such as these have highlighted the potential for image-based point clouds to offer low cost, objective and accurate methods for measuring vegetation structure and biomass.

The objective of this paper is to investigate the potential for point clouds generated from smartphone imagery to quantify surface and near-surface fuel structure. This paper will compare the repeatability of visual assessments using the OFHAG and image-based point clouds for assessing hazards in Australian bushfire fuel in Lowland Forest of eastern Victoria, Australia.

Specifically, this paper will assess:

- the variability between visual estimates of surface and near-surface fuel hazard and underpinning variables obtained by assessment teams using the OFHAG;
- the variability in metrics derived from image-based point clouds (Fuels3D method) with respect to cover and height of surface and near-surface fuels at both the plot and sample level, and derived surface fuel hazard rating;
- the agreement between metrics derived from the two methods; and
- the influence of differing smartphone models on metrics derived from image-based point clouds.

## 2. Materials and Methods

### 2.1. Study Area

**Figure 1:**
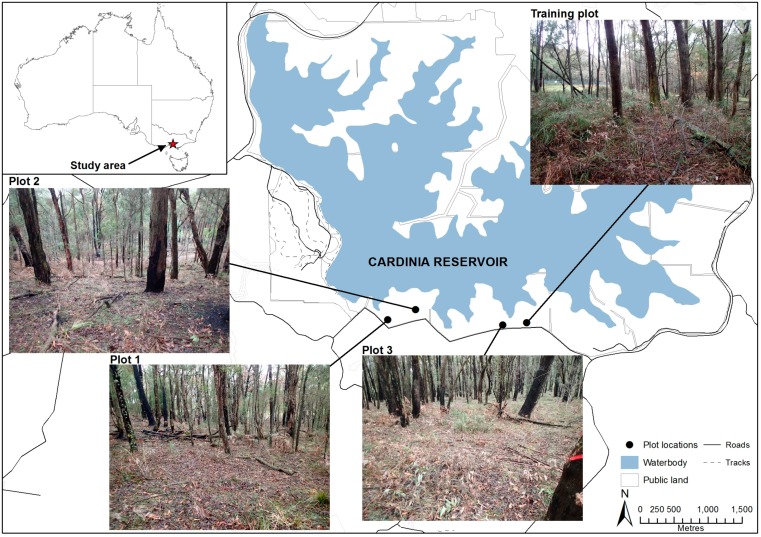
Location of the study area southeast of Melbourne, Australia, with the location and photos of study plots in Cardinia Reservoir (shaded blue).

The study area was located within the southern section of Cardinia Reservoir, approximately 49 km south-east of Melbourne’s CBD (Figure 1). Vegetation was characteristic of Ecological Vegetation Class (EVC) 6: Lowland Forest [49], with an overstorey of stringy and rough-barked eucalypts including Messmate stringybark *Eucalyptus obliqua*, Red stringybark *E. macroyhyncha*, and Narrow-leaf peppermint *E. radiata* s.l. The understory was dominated by native shrub (Common heath *Epacris impressa*, Prickly tea-tree *Leptospermum continentale*), graminoid species (Thatch saw-sedge *Gahnia radula*, Spiny-headed mat-rush *Lomandra longifolia*) and Austral bracken (*Pteridium esculentum*).

Three survey plots and one training plot were located within the study area (Figure 1). Plots 1 and 2 were located within an area subject to a prescribed burn on 28 April 2016. Plot 3 and the training plot were located within an area that was subject to a prescribed burn in 2012. 

### 2.2. Assessors and Visual Fuel Hazard Assessment

Assessments were undertaken by a total of 16 assessors, forming eight teams of two people. Each team recorded fuel hazard estimates and related data for Plots 1, 2 and 3 using the Overall Fuel Hazard Assessment Guide (OFHAG) [21]. Assessments were carried out on 15 and 21 July 2016 by personnel from various government agencies including the Victorian Country Fire Authority (CFA), Melbourne Water, Parks Victoria, Victorian Department of Environment, Land Water and Planning (DELWP), South Australian Department of Environment, Water, and Natural Resources (DEWNR), and Australian Capital Territory (ACT) Parks and Conservation Service, all of which were employed in positions relating in some way to fire management. The number of years of experience in assessing fuel hazard visually varied across assessors, ranging from less than one year to 30 years. Over 80% of assessors were currently active in collecting data relating to fuel hazard. Some assessors had previously undertaken formal training for fuel assessments, while others had learned “on the job”.

A brief training session was held at the training plot prior to undertaking the visual assessment in order to identify and agree on examples of fuel hazard for each layer and to encourage assessors to utilise the OFHAG consistently and appropriately. The training session also alerted assessors to potential sources of bias and confusion in the fuel hazard assessment process, and demonstrated the Fuels3D data collection method.

Assessors worked in pairs in order to produce a single set of data for the team for all components of the fuel assessment. Each individual assessor assessed each plot once, and, at any one time, a maximum of two teams were present within each plot. Interaction between teams was not encouraged during the assessment. The centre of each plot was clearly marked, and, as per the OFHAG, surface, near-surface and elevated fuel characteristics were assessed over a 10 m radius, while bark fuels were assessed over 20 m. Hazard ratings (Low, Moderate, High, Very High, Extreme) were assigned for each fuel layer, and data relating to the underpinning attributes were estimated following the OFHAG such as percent cover, height and proportion of dead material. Assessors were instructed to determine average litter depth by calculating the mean of five random measurements to the nearest millimetre using a ruler. Other fuel heights and percentage cover were estimated visually.

### 2.3. Image Capture

Images for the point clouds were captured using three different smartphone models loaded with the Fuels3D application (Table 1). Fuels3D is a beta application that has been developed by researchers at Royal Melbourne Institute of Technology (RMIT) University as a data collection tool for measuring fuel hazard and fire severity in the forest understorey [48]. Images were captured using a maximum of 11 megapixels, as programmed by the Fuels3D application.

**Figure 2:**
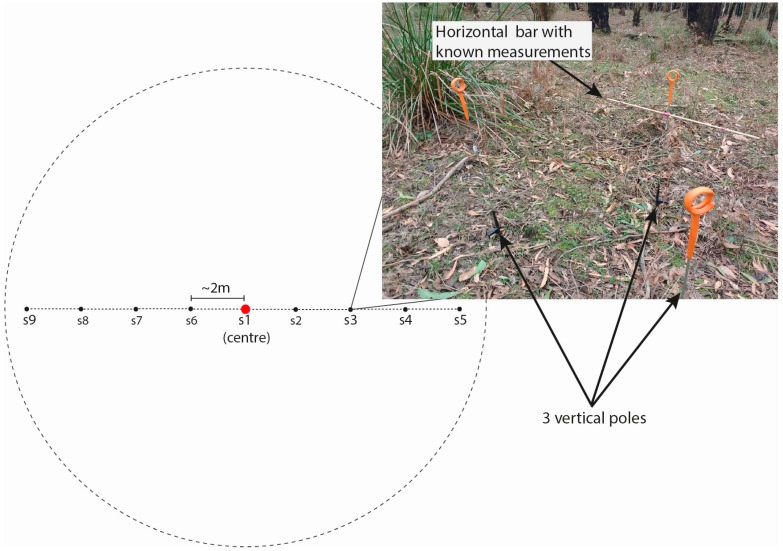
Diagram of transect and plot layout for image capture, showing the approximate location of samples (s1 to s9), and a photograph indicating frame set up.

Working in the same teams as for the visual fuel hazard assessment, assessors were instructed to capture imagery across 9 sampling locations (or “sub-plots”) spaced 2 m apart along a transect with a random bearing passing through the centre of each plot. Sample 1 was located at the marked centre of the plot, with two sets of 4 samples radiating away from the centre (Figure 2).

Samples were taken over a reference “frame” consisting of three separate vertical poles placed approximately 1 m apart in a triangular shape (Figure 2). A horizontal bar with known measurements marked was attached to one of the vertical poles. Assessors were instructed to take approximately 30 images per sample around the frame from approximately ground level to above the frame, in order to achieve a dome-like coverage of the sample.

### 2.4. Standardised Image Capture and Smartphone Model Comparison

An additional set of imagery was captured across the three plots on 4 August 2016. Imagery was captured using the same method in the preceding section; however, the same transect was sampled three times for each plot by the same assessor, once for each smartphone model utilised in the study. The purpose of this was to investigate potential differences in smartphone model performance with respect to the image-based point clouds generated.

### 2.5. Image-Based Point Cloud Generation

Images were collated for each plot, and the image matching procedure of Agisoft Photoscan Professional software version 1.2.6 (Agisoft LLC, Moscow, Russia) was used to construct a dense point cloud. High quality image matching was used to generate a sparse point cloud for each sample. This was followed by dense point cloud generation using the high accuracy setting and a mild depth filter. Images that were unable to be matched, or those that contained significant blur, sun-glare or obscurations were typically excluded from the point cloud, both manually and programmatically. Correct scale and orientation of the resulting point clouds was achieved via manual digitisation using a set of seven known target measurements on the frame, enabling the cloud to be rotated into object space. Features not considered surface or near-surface fuels such as tree stems were manually removed from the point clouds.

### 2.6. Metric Derivation

The OFHAG requires assessors to divide fuel into vertical fuel layers that are towards the bottom of the fuel profile, i.e., surface and near-surface fuels. The main difference between the two, according to Hines et al. [21], is that near-surface fuels may be comprised of living and dead material that is effectively in contact with the ground, but not lying on it, while surface fuels are lying on the ground and are primarily in a horizontal orientation. Surface fuels contain leaf litter, including leaves, twigs, bark and other fine fuels [21]. A method was thus developed using structure from motion (SfM) that aims to enable the separation of surface and near-surface fuel layers in point clouds.

Points were initially assigned to a 0.3 cm resolution voxel (i.e., a 3D pixel) space, with voxels considered filled when they contained at least one point. This voxel space was then segmented into contiguous segments with filled voxels considered part of the same segment where any of the sides or corners of neighbouring filled voxels touched. Points that fell within the segment containing the lowest filled voxel were initially attributed as surface points, while all other points were attributed as near-surface fuel points.

Hines et al. [21] do not explicitly mention the presence of live material in the surface fuel layer as per near-surface fuels, and, therefore, it has been inferred that surface fuels do not contain any living material. As this initial set of surface fuels included live vegetation, it was considered that some points had been misattributed. Further refinement of surface fuel points was then undertaken by taking into account point pixel colour. A greenness index value was calculated for each point based on its red, green and blue value using the following equation (as per [50]):

(1) $$Greeness=\frac{\left(2\times G-R-B\right)}{\left(2\times G+R+B\right)}$$

where R, G and B are the digital numbers of the red, green and blue channels, respectively. 

Similar greenness indices have been applied in the literature to assess the living status and condition of vegetation [51,52]. This particular index was chosen as it provides normalised RGB values between −1 and +1 based on the total scene illumination, where negative values typically represent soil and non-living material while positive values represent living matter [50]. Using this index, points originally identified as surface fuels with a positive greenness value were re-classified as near-surface fuels.

### 2.7. Point Cloud Normalisation

In order to derive metrics relating to surface and near-surface fuels, the height of each point above the ground surface is required to be determined, which requires an estimation of the soil surface. This was accomplished by filtering the surface fuel points to determine those points originating from the ground. This involved identifying the minimum surface fuel point within each cell of a 5 cm resolution, before interpolating the set of points to a higher 2 cm resolution grid via cubic convolution. These resolutions were chosen based on the high point density of the point clouds, and under the assumption that at least one ground point was visible in every 5 cm grid cell. The height of any surface fuel points occurring below this grid were then attributed to that collocated grid cell. Finally, subtracting the grid cell value at the location of each surface and near-surface fuel point from its initial height resulted in the creation of a normalised set of points for metric derivation.

### 2.8. Surface and Near-Surface Fuel Metrics

The OFHAG requires assessors to estimate surface fuel hazard through an estimation of average litter bed depth (or average fuel height) and percentage cover of fuel, where fuel height represents the distance between the mineral soil surface and the top of the litter bed [21]. The metrics of fuel height and cover were extracted from point clouds by attributing each surface fuel point to a cell within a 0.5 cm grid. A grid cell was considered to contain fuel if any of the points falling in that cell had a normalised height of greater than 0.5 cm. This allowed percentage cover to be calculated as the percentage of total cells that contained fuel. Fuel height for each cell was estimated for as the maximum normalised height within each cell.

Near-surface fuels are also assessed by estimating fuel height and percentage cover according to the OFHAG [21]. The height and cover of near-surface fuels were determined in the same manner as surface fuels. Unlike surface fuels, however, Hines et al. [21] also require the estimation of near-surface fuel that is dead. To determine the status of a cell, the mean red, green and blue values for all points within each 0.5 cm grid cell were determined. These mean values were then used to calculate a greenness value (Equation (1)) for each point. The percentage of dead fuel was calculated by dividing the number of cells with a negative greenness value by the total number of cells containing at least one near-surface fuel point.

The means of surface fuel height, surface fuel cover, near surface fuel cover and near surface percent dead across each of the nine samples were used as the plot level estimate. The mean of near-surface height was also calculated at the plot level. However, the mean of the maximum near-surface height of each sample was also calculated, as it was considered to more closely represent the observation of near surface height made by the participants during visual assessments.

### 2.9. Fuel Load and Hazard Score Calculations

Surface fuel load was calculated for both visually assessed and point cloud data by inputting estimates of surface litter depth (height) and percent cover obtained via the two methods into the following Equation (2) as per Wallace et al. [40]:

(2) $$Fuel\;load\;=\frac{1.46\;\times \;height\;\times \;cover}{\left(\pi \;\times \;10^{2}\right)+0.98}$$

The resulting fuel load estimates were converted to surface fuel hazard scores (Low, Moderate, High, Very High, Extreme) for each plot by using conversion tables provided in the fuel hazard assessment data sheet by the Department of Environment and Natural Resources, Adelaide, South Australia [53]. Although a similar conversion table is provided in the OFHAG [21], it is unclear how overlapping fuel load values in this table should be interpreted, and therefore it was not used in this study.

### 2.10. Analysis

Analysis of visual fuel hazard assessment measurements was limited to underpinning surface and near-surface attributes (i.e., percent cover, height), as these were the only attributes comparable with the data extracted from the image-based point clouds using Fuels3D. This analysis required the generation of single numeric inputs. Where assessment teams responded with a numeric range, the mid-point of the range was accepted. Where assessment teams responded with greater or less than estimates, a factor of 1 was added or subtracted to the given value.

Between team variability was assessed for each plot and assessment method by calculating coefficients of variation (CV, as percentage) and standard error of the mean (SEM) for each numeric variable. Agreement between measurements obtained by the two methods was similarly analysed using these statistics as well as the mean. The effect of variables other than assessment teams (plot, smartphone model) was assessed similarly by calculating the aforementioned measures of variability, with plot assessed at the sample level (Sample 1), and smartphone variability assessed at the site level.

## 3. Results

### 3.1. Point Cloud Properties

Assessment teams collected a total of 216 samples (photosets) across the three plots. Between 18 and 94 images were collected for each sample. A total of 81 samples (27 per smartphone model) were collected on the third day of assessment, with between 30 and 53 images collected for each sample.

**Figure 3:**
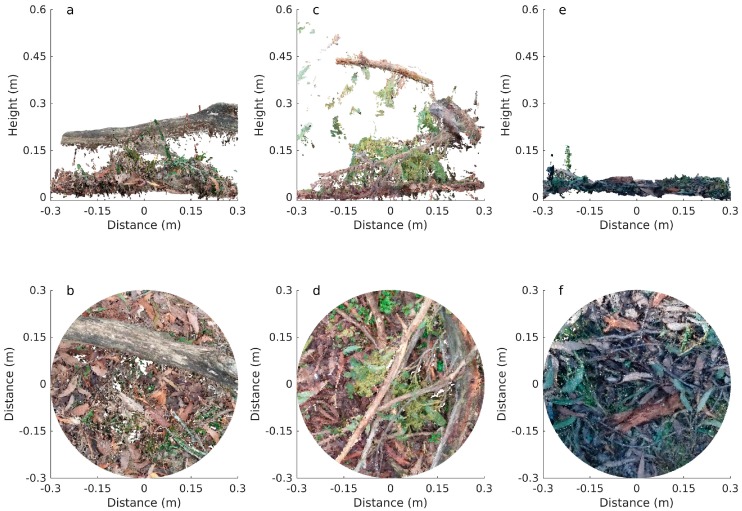
Example point clouds derived from the photosets captured in Plots 1, 2 and 3. (**a,b**) show a point cloud captured in Plot 1 with the Oppo phone, (**c**,**d**) show a point cloud captured in Plot 2 with the Motorola phone and (**e**,**f**) show a point cloud captured in Plot 3 with the Sony phone. Distance is measured from the plot centre, and height reflects estimated height above sea level.

Image matching and point cloud derivation was achieved for the majority of samples, resulting in the successful 3D reconstruction of surface and near-surface fuels (see Figure 3 for example). However, point clouds were unable to be derived for approximately 8% of samples, largely due to the affects of image quality (e.g., blur, sun-glare, etc.), or where there were insufficient images for the image-matching process.

### 3.2. Consistency between Smartphone Models

Little difference was observed at the site level between estimates of surface fuel attributes for the three phone cameras used. Mean surface litter height was fairly consistent between phone cameras, with a maximum difference of 1.5 mm observed between the Motorola and Sony, and the SEM almost equal for each camera (Table 2). Estimates of mean surface litter cover were also similar between phone cameras, with the largest difference observed between the Motorola (93.4%) and Sony (96.8%). Discrepancies between phone cameras were most noticeable in near-surface fuel attributes such as mean percent cover and mean maximum height, which ranged from 19.9% to 30.7% and 10.1 cm to 12.0 cm, respectively. However, the SEM observed for each phone camera was similar for near-surface mean maximum height measurements.

Overall, estimates derived from the Motorola’s imagery were least similar to the other two smartphone models and resulted in the highest SEM for half of the observed attributes. By comparison, mean values obtained from the Sony and Oppo were largely similar, particularly for surface litter cover (difference of 0.3%) and height (difference of 0.3 mm), and the cover of dead near-surface fuels (difference of 0.5%). 

### 3.3. Plot Level Variability between Assessment Teams

The magnitude of variation (as indicated by the CV) around any given mean varied for the underpinning surface and near-surface fuel attributes assessed at the plot-level (Table 3). Observer estimates from the visual assessment tended to be less variable (lower CVs) for surface litter percent cover and surface litter depth relative to other attributes, with CVs between 7.7 to 20 and 26.6 to 34.5 for these attributes, respectively (Table 3). Similarly, these two attributes varied little between teams’ point clouds, with the CV as low as 1.6 for surface litter cover in Plot 1 (Table 3). The amount of error (SEM) for these two attributes was also low for both methods.

Assessment teams varied widely in their visual estimates of all near-surface attributes, with CVs ranging from 41.0 (near-surface percent dead) in Plot 3 to 74.3 (near-surface average height) in Plot 2. Although the degree of variability in near-surface fuel attributes was lower using the Fuels3D method, variation was still evident. Variation was particularly high for mean maximum height and percent dead, with CVs ranging from 24.2 to 30.6 and 17.4 to 31.9, respectively (Table 3). Variation in mean near-surface height and cover was also evident, particularly within Plots 1 and 2, where CVs were high for these attributes.

### 3.4. Sample Level Variability between Assessment Teams

Point cloud measurements obtained from sample 1 demonstrated comparatively lower variability than that seen in visual counterparts (Table 4). Similar to the visual assessments, surface percent cover was the least variable parameter with CVs between 2.5 (Plot 3) to 3.3 (Plot 2) (Table 4). Variability in surface litter height was also low (CVs 14 to 18.3) compared to other attributes such as near-surface cover (CVs 24.5 to 30.1) and the percentage of dead near-surface fuels (CVs 18.7 to 21.3).

Near-surface height estimates varied widely between plots, particularly mean maximum height with CVs between 16.2 in Plot 1 to 61.5 in Plot 3.

Overall, Plot 3 recorded the greatest amount of collective variability across all attributes measured within sample 1. However, CV values for this plot were still lower than those obtained in visual assessments for most corresponding attributes (Table 3), with the exception of near-surface mean maximum height.

### 3.5. Agreement between the Two Methods

**Figure 4:**
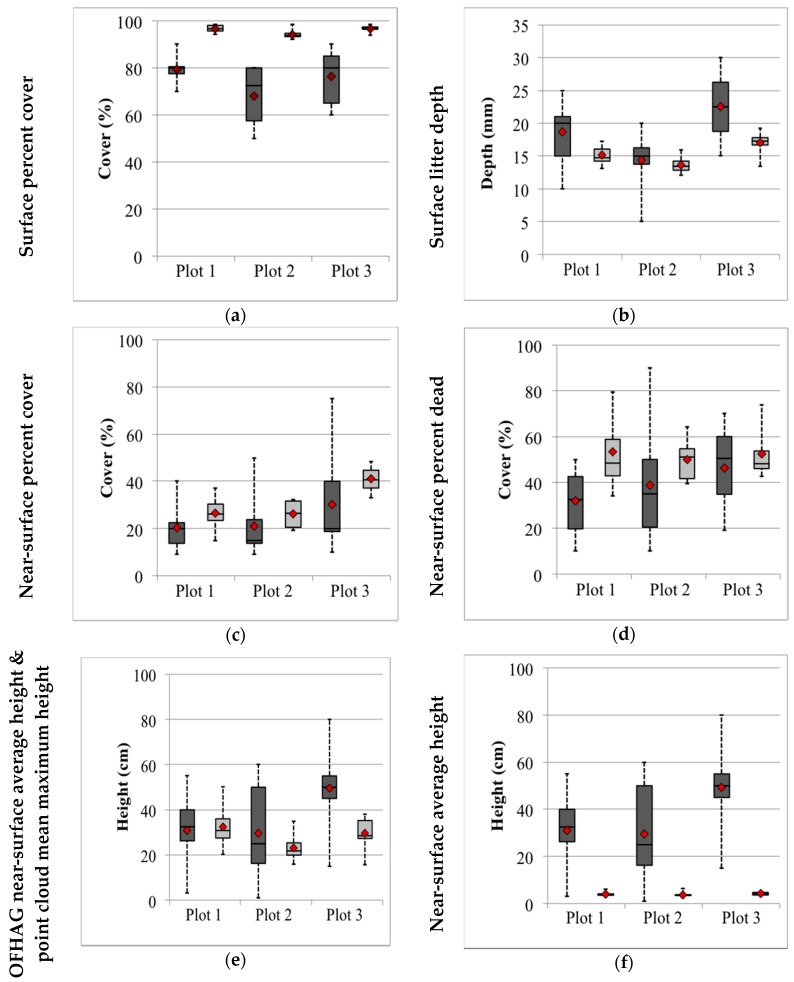
Boxplots of eight assessment teams’ estimates of surface and near-surface fuel attributes from visual assessments (dark grey), and image-based point clouds (light grey) across Plots 1, 2 and 3. Boxplots show the median and quartiles, minimum (lower whisker), maximum (upper whisker), with mean (red point) also shown. (**a**) surface percent cover, (**b**) surface litter depth, (**c**) near-surface percent cover, (**d**) near-surface percent dead, (**e**) OFHAG near-surface average height and point cloud mean maximum height, and (**f**) near-surface average height.

There were large differences between estimates of surface and near-surface fuel parameters between the two methods (Figure 4a–f). Mean surface litter cover values derived from point clouds were greater than corresponding visual estimates (Figure 4a), ranging from 94.3% to 96.7% compared to 68.1% to 79.4% in the visual assessments (Table 3). Conversely, estimates of mean surface litter depth were lower in Fuels3D than in visual estimates, with differences ranging from 0.8 mm (Plot 2) to 5.4 mm (Plot 3) (Figure 4b).

Where near-surface parameters were assessed, measurements resulting from the point clouds did not differ greatly for most corresponding visual estimates. Mean values of near-surface percent cover, for example, were fairly similar to their visually assessed counterparts for Plots 1 and 2 (Figure 4c); however, estimates from the point clouds were more precise than visual estimates. With respect to the percentage of dead near-surface fuels, the difference between mean values for both methods varied between plots, from 6.4% in Plot 3 to 21.5% in Plot 1 (Figure 4d). Point cloud measurements for this attribute were more precise than visual estimates for all plots; however, Plot 1 exhibited greater standard error for this attribute compared to its visual counterpart (Table 3).

There was some agreement between average height from the visual assessments and mean maximum height from the point clouds (Figure 4e), particularly within Plots 1 and 2, where differences were 1.3 cm and 6.6 cm, respectively. Mean height values differed greatly between the two methods though with point cloud estimates substantially lower than visual estimates in all plots (Figure 4f). The largest difference was recorded within Plot 3, where the average height estimated from point clouds was 45.2 cm lower than that estimated visually.

### 3.6. Fuel Hazard Scores

**Figure 5:**
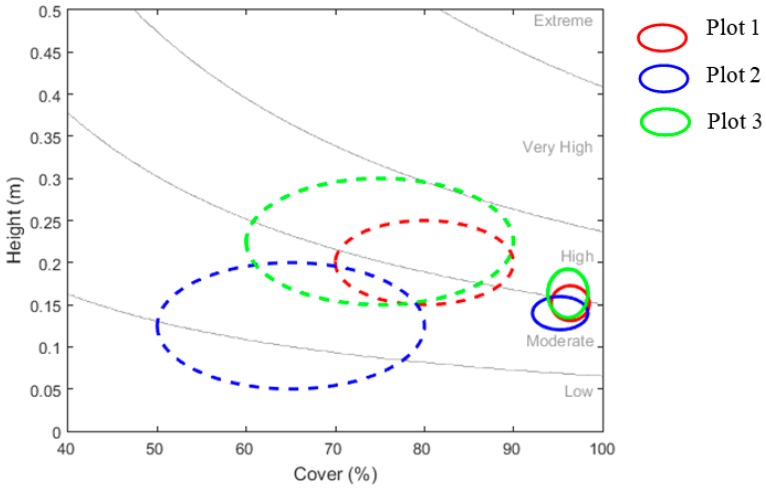
Isochrones showing the spread of surface litter height (*y*-axis) and percent cover values (*x*-axis) and resulting surface fuel hazard score from visually assessed metrics (dashed outline) and point cloud metrics (solid outline) across Plots 1, 2 and 3.

The difference between surface fuel metrics obtained via the two methods resulted in differences in the calculated surface fuel hazard score (FHS) for most of the plots. Visually estimated attributes within Plot 2 resulted in a FHS from “Low” to “Moderate”, while equivalent point cloud values obtained a single FHS of “Moderate” (Figure 5). Within Plot 3, the FHS calculated using visual estimates spanned three hazard categories, while the FHS from point cloud values spanned two categories. Plot 1 by comparison obtained the same calculated FHS range for both methods (“Moderate” to “High”). However, the precision of the hazard score obtained using point cloud metrics was greater (smaller isochrones) than that seen in the visual assessments for all three plots (Figure 5).

## 4. Discussion

This study found considerable variability between assessment teams for visual estimates of all surface and near-surface fuel components using the OFHAG. Subjectivity in visual assessments is well documented in the literature, with several studies demonstrating the inconsistencies that can arise between assessors [24,25,27,28]. The findings of this study are consistent with those documented by Watson et al. [24], which found significant assessor variability on fuel hazard scores and individual parameters such as percent cover and height for surface and near-surface fuels. By comparison, the lower variability observed in teams’ point clouds indicates that the Fuels3D method is capable of quantifying fuel attributes with greater repeatability than visual assessments. 

Few studies, if any, have assessed the variability of point cloud methods in conjunction with visual assessments; however, there is evidence to suggest that this relatively new photogrammetric technique is capable of accurately quantifying vegetation structure. For example, studies have demonstrated the capacity for point clouds to consistently and accurately quantify near-surface vegetation using Terrestrial Laser Scanning [35,41], while Wallace et al. [48] have demonstrated the potential for smartphone imagery to adequately reconstruct the 3D structure of grassland environments. Furthermore, a recent study conducted by Wallace et al. [40] using a handheld camera found a high correlation between destructively sampled biomass and vegetation volume derived from image-based point clouds in pasture (*r*^2^ = 0.78), dry grassy forest (*r*^2^ = 0.87) and lowland forest (*r*^2^ = 0.63). However, the characteristics of point clouds from teams’ smartphone imagery such as photographing technique, image arrangement and illumination conditions also introduce advantages and challenges to estimating surface and near-surface fuel attributes using this method.

Much of the variability that was observed in quantitatively measured attributes for the visual assessment, such as surface litter depth, and for most Fuels3D components are likely to be methods related. For example, the variability observed in assessors’ visual estimates of surface litter depth could be due to the interpretation of the sampling methods prescribed in the OFHAG and the subsequent approach undertaken by individual assessors. Five random samples of surface litter depth may have been insufficient to capture the variability of surface litter within the three plots, and in some cases it was observed that assessors “eyeballed” litter depth rather than taking physical measurements. Conversely, the transect sampling approach used in the Fuels3D method increased the number of samples obtained by each team from five point based samples (as prescribed in the OFHAG) to nine “sub-plots”, potentially explaining the lower variability seen here in surface litter depth (Table 3). The variability that was observed in point cloud estimates may also be methods related, as the user-determined placement of transects within the plots may have led some teams to inadvertently avoid areas of variable terrain (e.g., around large logs, small pits in the ground) and hence litter depth. Another possibility is that the height threshold used to derive surface metrics from the point clouds could have caused areas of the ground to be misattributed as surface fuels, thus resulting in an overestimation of surface litter cover compared to visual assessments (Figure 4a).

Variations between teams’ estimates of near-surface fuel attributes were considerably larger than that seen in surface fuels for both methods. With respect to the visual assessments, much of this variability is likely due to assessor subjectivity. For instance, high variability in visually assessed near-surface cover could be partly attributed to difficulties distinguishing between near-surface and elevated fuels, as assessors may have found it difficult to decide which fuel layer to allocate living and dead specimens of Austral bracken of varying heights. The potential for species such as Austral bracken to transition between different fuel layers is noted within the OFHAG [21] and has been identified as a potential source of variability in a previous study [24]. As bracken was largely absent from Plot 2 due to a recent planned burn, the variability seen here may instead relate to subjectivity of the OFHAG with respect to differentiating between surface and near-surface fuel layers. This may have caused differences in the way recently fallen leaves and mosses were treated by teams when estimating near-surface cover in this plot. Recently fallen living litter is likely to have been attributed as near-surface fuels in the Fuels3D method, thus resulting in a slight increase in mean values of near-surface fuel cover compared to the visual assessment (Figure 4c).

Although on average three times less variable than the visual assessments, differences in estimates of near-surface cover was still evident between teams’ point clouds. Mean cover was particularly variable within Plots 1 and 2 and could be explained by the placement of transects. For instance, clusters of near-surface fuels may have been overlooked by the placement of transects, and therefore the spatial variation in fuel cover across these plots may not have been fully captured by all teams. This issue was less pronounced in Plot 3, potentially as the cover of near-surface fuel was high and therefore more easily captured by the transects. Based on these results, it is considered that increasing the sampling density within environments where near-surface fuel cover is low or sporadically distributed could improve the variation seen in this attribute.

There was some disagreement between the proportions of dead near-surface material measured by the two methods, with mean values from point clouds up to 1.7 times greater than visual estimates. Furthermore, visual estimates were on average two times more variable than those from point clouds. This high variability could again be due to difficulties in assessors differentiating between surface, near-surface and elevated fuels using the OFHAG. For the Fuels3D method, the observed variation could partly be due to transect placement, as well as the method used to derive estimates of dead biomass from the point clouds. This method relies on pixel values of the smartphone imagery to estimate greenness, and therefore is vulnerable to the spectral stability of the smartphone cameras and lighting conditions in the field. Low light levels during the afternoon were found to cause substantial shadow in many assessors’ images, affecting the pixel values and thus the greenness of cells in the point cloud. Using smartphones with greater spectral stability could improve the precision of point cloud estimates, as could modifications to the camera either directly or indirectly through an externally mounted filter. This would enable different spectral properties to be captured, a technique which has proven effective in standard commercial-grade cameras modified to monitor vegetation health [52]. Nevertheless, the lower variability seen in the Fuels3D method for this attribute demonstrates the potential for this method to more consistently estimate the proportion of dead material than visual assessments.

The variability in near-surface height metrics was particularly high for both methods assessed. With respect to the visual assessments, this is considered to be an artefact of the way assessors perceived average layer height, as some teams may have inadvertently overlooked sub-dominant near-surface elements and therefore overestimated average height for this layer. The fact that differences between near-surface height estimates for the two methods were substantially lower when mean maximum height from the point clouds was considered provides further support for this. Furthermore, Gosper et al. [54] found that this “eyeballing” of vegetation height likely explained discrepancies in estimates obtained using visual fuel assessments within Western Australia. It is also a noted occurrence within the elevated fuel layer, with a future edition of the OFHAG likely to include instructions for assessors to record the average of both the top and bottom height of elevated fuels [55].

The Fuels3D method also showed variability in near-surface height estimates. In particular, the high variability seen in mean maximum height estimates indicates that improvements to image capture methods may be required. It is likely that the high variability seen in this attribute both at the sample and plot level was due to the technique of image capture employed by assessors, with low positioning of the smartphone causing the height of near-surface fuels to be truncated. A similar issue has been demonstrated in imagery obtained using UAV-mounted cameras, with view angle significantly affecting point-matching stability and vegetation reconstruction, resulting in increased error in canopy height estimates [56]. In respect to the Fuels3D method, it is believed that this issue could be overcome by providing additional instructions to assessors on adequate image capture technique.

### Implications for Management

One of the main aims of the OFHAG is to determine the level of fuel hazard present within the landscape in order for fire practitioners to assess the difficulty of controlling a bushfire [21]. As the allocation of hazard ratings for each of the fuel strata is based on the visual assessment of underpinning fuel attributes, it is reasonable to suggest that the subjectivity of such measurements between assessors could lead to variable assignment of fuel hazard. While it is suggested that a discrepancy of one hazard rating between assessors is inconsequential with respect to overall fuel hazard [21], much larger variation can occur. For instance, Watson et al. [24] found significant differences between assessment teams in all component hazard score variables, resulting in hazard ratings spanning across four levels, while Volkova et al. [30] found that the random allocation of surface fuel hazard ratings did not significantly differ to those that were visually assessed, indicating substantial variability in the assignment of hazard ratings by assessors. The results presented within this study suggest a similar occurrence, with hazard ratings calculated for surface fuels based on visually assessed metrics spanning up to three categories (Figure 5). The greater precision afforded by point cloud metrics by comparison resulted in more discrete surface fuel hazard scores. For instance, point cloud metrics resulted in a single surface fuel hazard rating of “Moderate” for Plot 2, while the calculated rating from visually assessed metrics spanned two hazard categories. The smaller isochrones in point cloud fuel hazard scores also suggest that measurements obtained using this method were able to determine hazard level with greater certainty than visual assessments.

Accurate measures of fuel hazard, fuel load and arrangement are required to inform a wide range of fire management activities such as assessing bushfire risk, planning fuel treatments, and managing smoke emissions [8,9,10,11,12]. The miscalculation of fuel hazard or fuel load due to inaccurate or inconsistent data can therefore have significant management implications. For example, underestimating fuel hazard could result in inaccurate estimates of lower bushfire risk and therefore exclusion of areas that should be prioritised for fuel reduction activities, potentially rendering properties and residents under-prepared. Conversely, overestimating fuel hazard could have implications with respect to inflating building costs within bushfire-prone areas [30]. Inaccurate and inconsistent estimates of fuel hazard could also lead to unreliable data for smoke dispersion models, predictions of greenhouse gas emissions and fire line intensity.

As destructive sampling was outside the scope of this study, the accuracy of the two methods to estimate fuel load cannot be determined. However, there is evidence to suggest that fuel loads derived from visually assessed hazard scores do not accurately reflect directly measured fuel loads. A recent study looking at forest fuel loads using the OFHAG found little correlation between visually assessed fuel hazard scores and destructively sampled biomass, with assessors tending to overestimate fuel hazard at low fuel loads and underestimate hazard at high fuel loads, regardless of fuel type [30]. Although it is important to note that assessments such as the OFHAG were not designed specifically for fuel load estimation, fire practitioners that are under resourced and facing time constraints may rely on the conversion tables provided by such assessments in order to obtain fuel load data [24,30]. The accuracy and consistency of such data is therefore imperative should it be used by fire and land managers in their decision-making regarding fire management practices.

Image-based point clouds have been shown to accurately quantify biomass in numerous recent studies [39,40,41,42,43]. These studies and the results presented within demonstrate the ability for the Fuels3D method to provide an efficient means of collecting consistent data on fuel structure and hazard that is less vulnerable to assessor subjectivity, with relatively little assessor training. Furthermore, the variability that was observed in the Fuels3D method could be overcome through user guidance and improvements to both the smartphone application and the equipment used. For instance, modifying the application so that it provides real-time quality assurance by analysing collected imagery and alerting the user when image quality is poor (e.g., blurry, obscured) could help improve the quality of the data derived from point clouds, while also fostering suitable image capture technique. Instructing users on appropriate camera positioning in order to capture the full vertical arrangement of fuels could also help to minimise variation in vegetation height estimates. Additional improvements may include smartphone camera modifications and the use of supplementary equipment, such externally mounted filters to improve spectral stability or introducing an object of known greenness to sample locations to standardise greenness values, thus improving estimates of dead biomass. This may also help to standardise camera settings between different smartphones, potentially improving the variability seen in certain phone models at the site level such as the Motorola, which performed differently to the other two models. The use of sensors in frame poles could enable precise estimates of ground level to be obtained, therefore improving the accuracy of surface cover measurements. Such sensors could also be used to provide a source of infield scale, and help in enabling automatic control point identification, therefore minimising post-processing time.

Further research on the Fuels3D method is currently taking place, such as testing of the technology across a more diverse range of fuel hazard landscapes, validating the metrics derived from point clouds with more precise data sources, and extracting information that is not currently captured within the OFHAG but which could prove useful in assessing fuel hazard, such as vertical connectivity. This continued research and development of techniques to improve the Fuels3D method would likely further enhance the application of such technology in the realm of bushfire management and research, and facilitate its complementary use with existing fuel hazard assessment practices such as the OFHAG.

## 5. Conclusions

This study has demonstrated the utility of image-based point clouds for consistently estimating surface and near-surface fuel attributes within lowland forest. The results of this study are consistent with the findings of others, and demonstrate that almost all visual estimates of fuel attributes using the OFHAG were vulnerable to subjectivity between assessors. While some variability in the Fuels3D method was apparent, it was substantially less than that seen in the visual assessments, both at the plot level and individual sample scale. Furthermore, most of the variability that was observed in the Fuels3D method can likely be reduced through improvements to both the methods and equipment used, while the majority of the variation seen in visual assessments is likely due to the inherent subjectivity of assessors and therefore may be difficult to overcome. This study demonstrates the capability of image-based point clouds and the Fuels3D method to efficiently collect objective estimates of fuel hazard, fuel structure and characteristics of individual fuel layers, and therefore its potential for use in a range of fire management practices including assessing bushfire risk, fire severity, and determining the efficacy prescribed burns.

## Figures and Tables

**Table 1:** Standard rear camera settings for three smartphone models used to capture imagery with Fuels3D.

Brand and Model	Screen Size and Resolution	Available Aperture	Sensor Size	Camera Megapixels
Sony Xperia Z1 compact	4.3′′	f/2.0	1/2.3′′	20.7
	720 × 1280 pixels			
Motorola Moto G (3rd Gen)	5′′	f/2.0	1/3.06′′	13
	720 × 1280 pixels			
Oppo F1	5′′	f/2.2	1/4′′	13
	720 × 1280 pixels			

**Table 2:** Mean (standard error) and coefficient of variation (CV) of surface and near-surface fuel attributes measured from point clouds at the site level using three smartphones: Motorola, Sony and Oppo.

Variable	Mean (s.e.)			CV (%)		
	Motorola	Oppo	Sony	Motorola	Oppo	Sony
Surface % cover	93.4 (1.7)	96.5 (1.0)	96.8 (0.8)	9.2	5.5	4.1
Surface height (mm)	12.9 (0.7)	14.1 (0.6)	14.4 (0.7)	28.9	23.2	26.8
Near-surface % cover	19.9 (2.3)	26.9 (2.2)	30.7 (3.0)	60.4	43.0	51.0
Near-surface % dead	65.6 (2.0)	67.2 (2.0)	66.7 (2.3)	15.9	15.2	18.2
Near-surface mean maximum height (cm)	10.8 (1.3)	12.0 (1.2)	10.1 (1.1)	60.3	50.7	54.2
Near-surface mean height (cm)	3.5 (0.4)	3.0 (0.2)	2.8 (0.1)	55.7	30.8	27.1

**Table 3:** Mean (standard error) and coefficient of variation (CV) between teams’ estimates of fuel attributes in Plots 1, 2 and 3 using the two methods. FHS, fuel hazard score (unshaded cells), PC, point cloud (shaded cells).

Variable	Plot 1		Plot 2		Plot 3	
	Mean (s.e.)	CV (%)	Mean (s.e.)	CV (%)	Mean (s.e.)	CV (%)
**Surface fuel**						
Litter depth (mm)	18.6 (1.8)	26.9	14.4 (1.8)	34.5	22.5 (2.1)	26.6
PC Litter depth (mm)	15.1 (0.5)	9.7	13.6 (0.5)	9.5	17.1 (0.6)	10.0
Litter cover (%)	79.4 (2.3)	7.7	68.1 (4.8)	20.0	76.3 (4.0)	14.8
PC Litter cover (%)	96.7 (0.5)	1.6	94.3 (0.7)	2.2	96.6 (0.6)	1.6
FHS	2.5 (0.2)	21.4	2.0 (0.0)	0.0	2.8 (0.3)	25.7
**Near-surface fuel**						
Cover (%)	20.5 (3.6)	50.3	21.1 (5.0)	67.2	30.0 (7.5)	70.7
PC Cover (%)	26.4 (2.4)	25.4	26.1 (2.0)	21.9	41.1 (2.0)	13.4
Height (cm)	31.0 (5.7)	51.7	29.5 (7.8)	74.3	49.4 (7.2)	41.4
PC mean max. height (cm)	32.3 (3.5)	30.6	22.9 (2.1)	26.5	29.5 (2.5)	24.2
PC mean height (cm)	4.0 (0.3)	21.9	3.8 (0.4)	29.2	4.1 (0.2)	14.3
Percentage dead	31.8 (5.4)	47.9	38.8 (9.0)	65.4	46.1 (6.7)	41.0
PC percentage dead	53.3 (6.0)	31.9	50.0 (3.1)	17.4	52.5 (4.0)	21.6
FHS	2.0 (0.3)	37.8	2.3 (0.2)	20.6	2.3 (0.2)	21.3

**Table 4:** Mean (standard error) and coefficient of variation (CV) between teams for fuel attributes measured using image-based point clouds within Sample 1 for each plot.

Variable	Plot 1		Plot 2		Plot 3	
	Mean (s.e.)	CV (%)	Mean (s.e.)	CV (%)	Mean (s.e.)	CV (%)
Surface fuel						
Cover (%)	96.3 (1.2)	3.0	94.3 (1.1)	3.3	96.1 (0.9)	2.5
Height (mm)	11.6 (0.7)	15.3	11.9 (0.6)	14.0	16.2 (1.1)	18.3
Near-surface fuel						
Cover (%)	13.0 (1.3)	24.5	22.9 (2.0)	25.2	31.4 (3.6)	30.1
Cover dead (%)	44.7 (3.5)	19.3	63.3 (4.8)	21.3	61.3 (4.3)	18.7
Mean maximum height (cm)	8.7 (0.6)	16.2	10.8 (0.9)	22.3	21.2 (4.9)	61.5
Mean height (cm)	2.6 (0.1)	11.3	2.8 (0.2)	19.7	3.5 (0.3)	21.3
